# Journal of Pharmacy and Bioallied Sciences

**DOI:** 10.4103/0975-7406.72126

**Published:** 2010

**Authors:** Mohd. Aqil

**Affiliations:** Editor, JPBS, Department of Pharmaceutics, Jamia Hamdard (Hamdard University), New Delhi - 110 062, India. E-mail: editor@jpbsonline.org


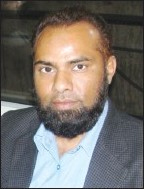


I hereby present our esteemed readers Vol. 2, Issue 4, 2010, of the *Journal of Pharmacy and Bioallied Sciences* (JPBS). JPBS was launched in the last quarter of the previous year with an aim to serve as a medium for high-quality research and review articles apropos to all aspects of pharmaceutical and allied biosciences. The journal is growing in stature with every issue with critical acclaims and encouraging reviews. The previous issues have set the tone and tenor for the present and forthcoming issues.

With the advent of new disciplines in pharmaceutical sciences and pharmacy practice including nanotechnology and pharmacovigilance, many new possibilities are on the offer for biomedical scientists. Both these branches are gaining ground and are finding escalating applications in their respective realms. Nanotechnology in particular has got wider acceptance in almost every walk of life including diagnostics and therapeutics. Investigations are currently under way on novel and precise nanotechnology based diagnostic tools and therapeutic devices such as nanochips and nanobots. Many such gadgets will see the light of the day in the ensuing times. Mankind will be the beneficiary of all these advancements in health sciences.

As for pharmacovigilance, the clinical trials conducted by pharmaceutical manufacturers are never exhaustive and cannot identify the delayed and very rare adverse drug reactions which might appear on chronic use of pharmaceuticals and medical devices. Hence, pharmacovigilance studies are the need of the hour for constant monitoring of adverse effects in hospital and community settings.

The present issue of JPBS presents an assortment of papers on contemporary topics viz. nanotechnology, fungal infections in children, neuropharmacological safety of jigrine, reversibility of asthma and COPD, orphan drug development, transdermal drug delivery, periodontitis and pharmacovigilance, etc. The issue also carries reports on new analytical methods developed for quantification of the active constituents in bulk and pharmaceutical formulations. I thank all the authors for their valuable contributions and look forward to opportunities to serve them in future.

At JPBS, we have a team of dedicated editors well advised and complimented by a diverse and competent editorial board of international magnitude. The editorial and technical staff at JPBS is working overtime to maintain the surging quality of the journal. We remain open to constructive criticism and healthy suggestions by our readers in further improvement and make over of the journal.

No journal can achieve greater heights without the constant patronage of its readers. We thank with gratitude the onerous support by our readers and institutions which have subscribed to our online and/or print issues. Nevertheless, I request to our existing patrons to recommend subscription of JPBS to their fellow professionals and institutional libraries to cater the scientific pursuit of all concerned.

I must laud the efforts of our esteemed reviewers who have devoted their precious time in critical review of the manuscripts submitted to journal. Without their support we would not have been able to publish quality papers in the JPBS. We will continue to seek their uninhibited support to future assignments.

Last but not the least, I thank our publishers, Medknow Publications, for their untiring efforts in designing, typesetting, copyediting, and improving the overall quality of the journal.

I close this piece with renewed solicitation to pharmaceutical and allied scientists to contribute quality research and review articles on topics of current interest and provide an opportunity to JPBS to disseminate their ideas and innovations to the scientific community across the globe.

